# Clinical efficacy and safety evaluation of traditional Chinese medicine for nourishing yin and Replenishing qi in combination with PD-1/PD-L1 inhibitors in the treatment of NSCLC patients: a meta-analysis

**DOI:** 10.1093/toxres/tfaf013

**Published:** 2025-01-26

**Authors:** Lijun Pan, Xueyan Wang, Fengxi Long, Amei Tang

**Affiliations:** Department of Medical Affairs, The First Affiliated Hospital of Guizhou University of Traditional Chinese Medicine, 71 Baoshan North Road, Yunyan District, Guiyang Guizhou 550001, China; The First College of Clinical Medicine, Guizhou University of Traditional Chinese Medicine, No. 4 Dongqing Road, Huaxi District, Guiyang Guizhou 550005, China; Development Planning Division, Guizhou University of Traditional Chinese Medicine, No. 4 Dongqing Road, Huaxi District, Guiyang Guizhou 550005, China; Respiratory Medicine, The First Affiliated Hospital of Guizhou University of Traditional Chinese Medicine, 71 Baoshan North Road, Yunyan District, Guiyang Guizhou 550001, China

**Keywords:** traditional Chinese medicine, replenishing qi and nourishing yin, PD-1/PD-L1 inhibitors, non-small cell lung cancer, chemotherapy, combination therapy

## Abstract

To examine the therapeutic effectiveness and safety of traditional Chinese medicine in conjunction with PD-1/PD-L1 inhibitors for nourishing yin and replenishing qi in patients with non-small cell lung cancer. A systematic search was conducted across seven electronic databases, namely PubMed, Cochrane Library, Excerpt Medica Database, Web of Science, Chinese Biomedical Literature Database, China National Knowledge Infrastructure, and Wan-fang Database, to identify eligible studies from 2,000 to 2,023. This study includes a total of 14 randomized controlled clinical trials, with 514 patients in the TCM combo therapy group and 506 patients in the control group. The meta-analysis demonstrated the efficacy of combining TCM in oral and injectable forms with PD-1/PD-L1 inhibitors, with or without chemotherapy, in enhancing objective response rate, disease control rate, and quality of life in patients with NSCLC. Additionally, this combination therapy improved the proportion of CD3 + T cells and CD4 + T cells, as well as the ratio of CD4+/CD8 + T lymphocytes. The group receiving combined treatment with TCM successfully decreased the expression of the tumor marker CYFRA21-1. The group receiving combination therapy with TCM decreased the probability of experiencing adverse effects such as nausea, leukopenia, thrombocytopenia, and exhaustion in patients. Additionally, this treatment did not place additional strain on the liver and kidney functions. The integration of TCM techniques that nourishing yin and replenishing qi with PD-1/PD-L1 inhibitors greatly enhances the clinical effectiveness and safety of treating NSCLC. Additionally, the combination of Chinese and Western drugs improves the efficacy of neoadjuvant chemotherapy in NSCLC patients.

## Introduction

Lung cancer exhibits the greatest occurrence and fatality rate among all types of cancer globally ^1^. Approximately 80%–85% of individuals diagnosed with lung cancer are found to have non-small cell lung cancer, which encompasses several primary subtypes including adenosquamous, big cell, adenocarcinoma, and squamous cell carcinoma^2^. Non-small cell lung cancer is characterized by the absence of usual symptoms during its initial stages, leading to the majority of patients being diagnosed with advanced illness^3^. The National Comprehensive Cancer Network (NCCN) released the 2nd version of the NCCN Clinical Practice Guidelines in Oncology: Non-Small Cell Lung Cancer in February 2023^4^. This edition emphasizes diagnostic principles, the diagnosis and treatment of different types of lung cancers, and the use of immunotherapy, among other topics. Immunotherapy utilizes immune checkpoint inhibitors (ICIs) as a common method for treating non-small cell lung cancer (NSCLC) in clinical settings. Specifically, monoclonal antibodies targeting programmed cell death receptor 1/programmed cell death receptor ligand 1 (PD-1/PD-L1) have demonstrated enhanced survival rates in patients with lung cancer^5^. At present, the standard initial treatment for advanced lung cancer is either PD-1/PD-L1 monotherapy or PD-1/PD-L1 combination chemotherapy. Although ICI stimulates the immune system to fight against tumors, it also causes many harmful consequences known as immune-related adverse events (irAEs).^6^^,^^7^

The adverse effects (irAEs) encompass a range of medical conditions, such as heart injury, immune-mediated pneumonitis, liver injury, gastrointestinal toxicity, renal injury, skin and mucosal toxicity, endocrine toxicity, and hypothyroidism^8^^,^^9^. Research has indicated that irAEs are present in 70% to 90% of patients who receive immune checkpoint inhibitors (ICIs), and severe irAEs occur in 10% to 15% of cases^10^. Additionally, these reactions can result in fatalities in 1.3% of patients^11^. The occurrence of any grade of irAEs in lung cancer patients treated with ICIs ranges from 24% to 38%. Additionally, there is a possible connection between irAEs and disease management as well as extended survival in lung cancer patients.^12–16^ A study^17^ conducted by demonstrated that the occurrence of all levels of immune-related adverse events (irAEs), particularly severe levels (grades 3–5), was more frequent in patients who received CTLA-4 inhibitors compared to those who received PD-1/PD-L1 inhibitors. A study^18^ analyzed a group of 1,022 patients who had stage III-IV lung cancer and were treated with PD-1/PD-L1 inhibitors. Out of these patients, 577 experienced irAEs, resulting in an overall incidence rate of 56.5%. Furthermore, the suboptimal effectiveness of ICIs in certain individuals with advanced lung cancer may be attributed to the emergence of immunological tolerance or the activation of the immune system^19^. Simultaneously, immune checkpoint inhibitors can induce severe adverse effects including endocrine dysregulation, immunological pneumonia, hypothyroidism, and skin damage.^9^ Additionally, a significant number of patients continue to experience unfavorable results with ICIs. The current study is focused on how to enhance the effectiveness of ICIs for the benefit of patients.

NSCLC is classified as “pulmonary retention,” “cough,” and “lung flaccidity” in traditional Chinese medicine. According to the theory of Traditional Chinese Medicine (TCM), the development of NSCLC is strongly linked to a lack of positive life energy (qi) and the invasion of harmful toxins. Patients with long-term illnesses often experience a depletion of their yin essence. The diagnosis of advanced lung cancer in clinical settings is also based on the deficiency of qi and yin. Therefore, the treatment approach for this disease should focus on replenishing qi, nourishing yin, and moisturizing the lungs.^20–22^ In traditional Chinese medicine, the accumulation of substances is believed to be caused by a lack of positive qi, allowing harmful qi to take hold. The concept of “positive qi” is considered to be the primary indicator of immunity in traditional Chinese medicine, which aligns with the use of immunological drug therapy in modern medicine.^23^^,^^24^ Chinese medicine exhibits holistic, systemic, and multi-targeting properties in its approach to preventing and treating tumors. It enhances the internal environment of the body, boosts immune function, and exerts an anti-tumor effect through the autoimmune system^25^^,^^26^. This aligns with the concept of using immunological drugs for tumor treatment. Modern pharmacological research findings indicate that nourishing yin and replenishing qi Traditional Chinese Medicine (TCM) possess immunomodulatory effects^27^^,^^28^. These effects can influence the body’s immune cells, contribute to the anti-tumor immune response, counteract the immune evasion of lung cancer, suppress the metastasis of tumor cells, and ultimately serve the goal of tumor treatment^29^. Hence, the combined immunotherapy of nourishing yin and replenishing qi in TCM has the capacity to enhance the responsiveness of patients with NSCLC to immunotherapy, enhance the clinical effectiveness of immunotherapy, and extend patient survival.

We conducted a systematic review and meta-analysis to examine the therapeutic effectiveness and safety of combining nourishing yin and replenishing qi traditional Chinese medicine (TCM) with PD-1/PD-L1 inhibitors, vs using PD-1/PD-L1 inhibitors alone. The studies included both types of combination chemotherapy and no combination chemotherapy. They were presented in a subgroup format to support the clinical use of nourishing yin and replenishing qi TCM combined with PD-1/PD-L1 inhibitors for treating NSCLC.

## Materials and methods

The protocol for this systematic review was registered on INPLASY with the unique ID number INPLASY202360049 and may be accessed in its entirety at inplasy.com. (https://inplasy.com/inplasy-2023-6-0049/).

### Eligibility criteria and outcome measures

The conditions for membership, as determined by the PICOS acronym^30^, were as follows: Participants: 1) Patients who have been diagnosed with non-small cell lung cancer (NSCLC) based on pathology, diagnostics, and imaging, following the Clinical Practice Guidelines for Molecular Pathology Testing of Non-Small Cell Lung Cancer^31^ and the NCCN Clinical Guidelines for the Management of Non-Small Cell Lung Cancer^4^. 2) There were no limitations based on gender, race, or country. 3) Patients willingly provided their signature on a written informed consent form, demonstrating a comprehensive comprehension of the study. 4) The study received approval from the hospital ethics committee.5) Patients with comorbidities that could potentially impact the experimental outcomes were excluded.6) Non-compliant patients were omitted from the study.

I (Intervention): This study includes randomized clinical trials (RCTs) that examined the effects of combining nourishing yin and replenishing qi traditional Chinese medicine with PD-1/PD-L1 inhibitors. There are no limitations regarding the combination or absence of chemotherapy, but combining nourishing yin and replenishing qi traditional Chinese medicine must be administered orally or via injection, not externally.

C (Comparison): The control group of patients with NSCLC were treated with PD-1/PD-L1 inhibitor regimens, either alone or in combination with chemotherapy, without any limitations.

O (Outcome): This study aims to evaluate the clinical effectiveness and safety of combining nourishing yin and replenishing qi Traditional Chinese Medicine (Oral or injection) with PD-1/PD-L1 inhibitors.

S (Study design): This study was conducted using a randomized controlled clinical trial design.

The following criteria were used to reject studies: (i) non-randomised controlled trials, (ii) studies with incomplete outcomes, and (iii) studies with insufficient data. The primary outcomes assessed in this study were three efficacy indicators: short-term and long-term clinical outcomes, as well as adverse drug reactions (ADRs), based on the criteria established by the World Health Organization (WHO) and the Response Evaluation Criteria in Solid Tumors (RECIST). (1) Immediate clinical results: Short-term tumor remission encompasses several outcomes, including complete remission (CR), partial remission (PR), stable remission (SD), progressive remission (PD), overall response rate (ORR), and disease control rate (DCR).

The outcome measures consist of short-term clinical efficacy, which is measured by the ORR and DCR. Other measures include adverse drug reactions (ADRs), tumor markers such as CA125 and CYFRA21-1, T-lymphocyte subsets including CD3+, CD4+, and CD4+/CD8+, and post-treatment Karnofsky Performance Status (KPS) scores.

### Search strategy and study selection

Two researchers independently conducted systematic literature searches in worldwide databases (Cochrane Library, PubMed, EMBASE, and Web of Science) as well as Chinese databases (CBM, CNKI, and Wan-fang Database) to identify relevant papers from 2,000 to 2,023. We conducted a simultaneous search for terms in all three orientations. The initial approach is nourishing yin and replenishing qi TCM, encompassing Traditional Chinese Medicine (TCM), Proprietary Chinese Medicine (pCm), decoction, capsule, fang, pill, injection, and granule. The second category is non-small cell lung cancer, which encompasses NSCLC, non-small cell lung cancer, and non-small cell carcinoma. The third category is PD-1/PD-L1 inhibitors, which consist of PD-1 inhibitors, PD-L1 inhibitors, cemiplimab, nivolumab, pembrolizumab, toripalimab, camrelizumab, tislelizumab, penpulimab, sintilimab, atezolizumab, avelumab, and durvalumab. Chinese search involves the utilization of keywords in Chinese databases. All the keywords in each direction are simultaneously inputted into the algorithm together with all the keywords in the other two directions for the initial search. The search formula is (((Traditional Chinese medicine) OR (Chinese medicine combined)) AND (PD-1 or PD-L1 inhibitors) AND (Non-small cell lung cancer), (((Traditional Chinese medicine) OR (Chinese medicine combined)) AND (Non-small cell lung cancer)) AND (Pembrolizumab), (((Traditional Chinese medicine) OR (Chinese medicine combined)) AND (Non-small cell lung cancer)) AND (Atezolizumab), etc. The title and abstract were assessed separately, and subsequently, the complete text of the pertinent literature was examined to ascertain suitability. The evaluation of Traditional Chinese Medicine (TCM), whether it is nourishing yin or replenishing qi, relies on analyzing the content and efficacy of the formula. Any inconsistencies were addressed with a third researcher. Furthermore, the authenticity of the original report and the sources cited in prior evaluations were verified.^32^

### Data extraction

The study extracted various characteristics related to the study and participants, such as the first author, publication year, study type, sample size, average age of participants, gender distribution, details of the Traditional Chinese Medicine (TCM) intervention (including dosage and duration), method of administration, details of the PD-1/PD-L1 inhibitor regimen (including dosage and cycle), details of the chemotherapy regimen (including dosage and cycle), and outcome measures. Consensus was reached to overcome disagreements.

### Quality assessment and evidence level

The quality of the studies was assessed using the Cochrane risk of bias tool Review Manager 5.4. The review criteria encompassed seven domains, namely random sequence generation, allocation concealment, blinding of participants and personnel, blinding of outcome assessments, inadequate outcome data, selective reporting, and additional sources of bias. The studies included in the analysis were evaluated using three categories: low, uncertain, and high risk of bias.

### Statistical analyses

The statistical analysis was conducted using Review Manager 5.4 and r4.2.2 software. The primary outcome measures were reported as risk ratios (RR) and standardized mean differences (SMD), along with their corresponding 95% confidence intervals (CIs). A p-value of less than 0.05 was deemed statistically significant. The Cochrane Q test and I^2^ statistics were employed to evaluate the presence of heterogeneity among studies, with a significance level of *P* ≤ 0.1 or an I^2^ value greater than 50% suggesting statistical heterogeneity. In the absence of statistical heterogeneity, fixed effects models were employed to compute the outcomes. Alternatively, a random-effects model was employed using the DerSimonian and Laird technique. In order to prevent the exaggeration of effects, studies that had no recorded incidents were included. Funnel plots were employed to assess publication bias if the number of research reporting consistent findings exceeded 10. Sensitivity analyses were conducted to assess the impact of individual studies on the aggregated findings by systematically excluding one study at a time from the overall analysis. Subgroup analysis was conducted based on whether the combination involved chemotherapy or not.

## Results

### Literature search and study characteristics

Initially, 1,596 papers were found, and after a selection process, 14 studies with 1,020 patients were included (Supplementary Fig. 1). The final exclusion of 1,299 articles after reading was due to various reasons: 1) The participants were non-human animals, specifically rats, mice, rabbits, and so on. 2) The essay in question was a critique, rather than a clinical trial. 3) The trial was not conducted using randomization and control groups. 4) The study did not specify the type of Traditional Chinese Medicine (TCM) employed, or the TCM used was not nourishing yin and replenishing qi TCM. 5) The trial does not provide explicit information regarding the specific type of immunosuppressant that was utilized. 6) The absence of a typical indicator grading system disallowed the inclusion of the data in the meta-analysis group. A total of 14 randomized controlled clinical studies were completed in China. Out of these, 10 trials utilized oral Chinese medicine, whereas 4 trials employed commercially accessible injectable Chinese medicine (Table 1).

**Table 1:** Features of a randomised controlled clinical trial of TCM in combination with PD-1/PD-L1 inhibitors in NSCLC(*n* = 1,020).

First author (yr)	Design	Sample size (M/C n)	Age (CT/C ye ars)	Sex (Male and Female n)		Combination therapy group intervention (Dosage and duration)	Control group intervention (Dosage and duration)	Outcome measures
				Combination therapy group	Control group			
Hao YJ 2023^36^	RCT	43/43	(54.16 ± 8.42)/(55.06 ± 8.36)	30/13	33/10	Zhengyuan capsule, oral, 4 capsules/dose, tid, for a total of 12 wk； Atezolizumab + conventional chemotherapy regimen	Atezolizumab + conventional chemotherapy regimen Atezolizumab and carboplatin, ivgtt, day 1 Paclitaxel, ivgtt, day 1 and day 8 21 days/cycle, for 4 cycles	O1,2,3,4
Wang S 2023^37^	RCT	49/49	(60.88 ± 6.77)/(59.51 ± 6.54)	26/23	22/27	Zhengyuan capsule, oral, 4 capsules/dose, tid, for a total of 3 mo； Pembrolizumab	Pembrolizumab, 2 mg·kg^−1^, ivgtt, q3w, for a total of 3 mo	O1,2,3
Chu SQ 2022^38^	RCT	60/60	(54.03 ± 1.61)/(51.02 ± 1.76)	36/24	33/27	Aidi injection, 50 mL/d, ivgtt,qd, for a total of 6 wk； Pembrolizumab + conventional chemotherapy regimen	Pembrolizumab + conventional chemotherapy regimen, Pembrolizumab,2 mg·kg^−1^, ivgtt, q3w, for a total of 6 wk	O1,3,4,5
Wang Y 2023^39^	RCT	50/51	(49.63 ± 8.97)/(49.67 ± 9.52)	33/17	37/14	compound Kushen injection，20 mL + 250 mL 0.9% NaCl dilution, ivgtt, qd for 21 days, 7 days pause, for 4 cycles; Camrelizumab + AP regimen	Camrelizumab + AP regimen Camrelizumab，3 mg/kg，iv，d1; pemetrexed, 500 mg/m^2^, ivgtt, d1; carboplatin, 300-400 mg/m^2^, dissolved in 500 mL saline, protected from light, ivgtt. 21 days/cycle, for 4 cycles	O1,2,3,4
Zhen XY 2022^40^	RCT	40/40	(60.83 ± 4.94)/(59.05 ± 4.28)	18/22	16/24	Baihe Gujin Decoction, 400 mL/time, bid, 21 days/cycle, total 6 cycles; Camrelizumab and PP regimen	Camrelizumab +PP regimen Camrelizumab,200 mg, ivgtt, d1 pemetrexed,500 mg/m^2^, ivgtt, d1 cisplatin,75 mg/m^2^, ivgtt, d1 21 days/cycle, for 6 cycles	O1,2,3,4
Li X 2022^41^	RCT	48/43	42 ~ 82 (62 ± 8.83)	/	/	Shashen Maidong Soup, Oral, 14 days/cycle, for 2 cycles; Sintilimab + conventional chemotherapy	Sintilimab + conventional chemotherapy Sintilimab 200 mg + 100 mL 0.9%NaCl, ivgtt, 21 days/cycle, for 2 cycles	O1,4
Chai HH 2022^42^	RCT	30/30	(65.60 ± 3.53)/(65.40 ± 3.76)	18/12	17/13	Compound Shou Gong San, 5 g/d, bid, 21 days/cycle, total 4 cycles; Camrelizumab	Camrelizumab, 200 mg/time, ivgtt, 21 days/cycle, total 4 cycles	O1,3,5
Zhang C 2022^43^	RCT	20/20	(65 ± 12)/(65 ± 11)	10/10	12/8	Zhengyuan capsule, oral, 4 capsules/dose, tid, 21 days/cycle, for 4 cycles； Sintilimab+PP regimen	Sintilimab+PP regimen/Paclitaxel, cisplatin pemetrexed,500 mg/m^2^, ivgtt, d1 Paclitaxel, 135 mg/m^2^, ivgtt, d1 cisplatin, AUC = 5, ivgtt, d1 21 days/cycle, for 4 cycles	O1,3,4
Liu MZ 2022^44^	RCT	30/30	(65.28 ± 4.63)/(66.11 ± 5.28)	17/13	19/11	Compound Cushing’s injection, 30 mL + 500 mL 5% glucose solution, ivgtt, qd for 12 wk; Atezolizumab+Carboplatin, etoposide	Atezolizumab+Carboplatin, etoposide Atezolizumab, 1,200 mg, ivgtt, day 1 Carboplatin, etoposide, ivgtt,day 1 for 12 wk.	O1,2,4

(*Continued*)

**Table 1:** Continued

First author (yr)	Design	Sample size (M/C n)	Age (CT/C ye ars)	Sex (Male and Female n)		Combination therapy group intervention (Dosage and duration)	Control group intervention (Dosage and duration)	Outcome measures
				Combination therapy group	Control group			
Chen TQ 2022^45^	RCT	30/30	(60.13 ± 12.40)/ (64.67 ± 9.28)	18/12	19/11	Astragalus polysaccharide injection, 250 mg + 500 mL 5% glucose solution, ivgtt,qd, d1-d10; Camrelizumab, Apatinib mesylate tablets	Camrelizumab, 200 mg, ivgtt, day 1,q2w Apatinib mesylate tablets, 250 mg, qd, half hour after meal 14 days/cycle, total 2 cycles	O1,3,4,5
Zhang ZT 2022^46^	RCT	30/30	(63.06 ± 3.503)/(63.50 ± 3.048)	19/11	16/14	Self-formulated Jianpi Chushi formula, bid, for a total of 6 wk; Sintilimab+PP regimen/Paclitaxel, cisplatin	Sintilimab+PP regimen/Paclitaxel, cisplatin pemetrexed,500 mg/m^2^, ivgtt, d1 Paclitaxel, 100 mg/m^2^, ivgtt, d1,d8 cisplatin,0.4 g/m^2^, ivgtt, d1, q3w, 21 days/cycle, for 2 cycles	O1,4,5
Liu FL 2022^48^	RCT	28/25	(41.63 ± 12.57)/ (43.47 ± 11.63)	16/12	14/11	Xiaoyan soup,150 mL/dose, bid, 14 days/cycle, total 3 cycles Nivolumab+Pemetrexed，Carboplatin/ Paclitaxel, Carboplatin	Nivolumab+Pemetrexed，Carboplatin/ Paclitaxel, Carboplatin Nivolumab, 3 mg/kg,ivgtt, day 1,q2w Pemetrexed,500 mg/m^2^, ivgtt, d1 Paclitaxel,130 mg/m^2^, ivgtt, d1,d8 Carboplatin,500 mg/m^2^, ivgtt, d1, 21 days/cycle, total 3 cycles	O1,3,5
Wang XY 2021^19^	RCT	28/27	(65.07 ± 4.83)/(63.74 ± 4.53)	17/11	15/12	Shenqi Yifei soup, bid, for a total of 8 wk Nivolumab	Nivolumab, 3 mg/kg,ivgtt, day 1,q2w,for a total of 8 wk	O1,2,3,5
Mi SC 2021^47^	RCT	28/28	66(56,78)/66(55.72)	14/14	16/12	Xuanfei Fuzheng Kangai soup, 125 mL/dose, bid, for a total of 3 mo; Sintilimab+Pemetrexed	Sintilimab, 200 mg + 100 mL 0.9% NaCl, ivgtt, Pemetrexed,500 mg/m^2^, ivgtt, d1, 21 days/cycle, total 4 cycles	O1,3,4

Abbreviations: bid—twice daily; d—day; ID—intravenous drip; ivgtt—intravenous; n—number; O—outcome, O1—clinical effectiveness; O2—tumour markers and correlates; O3—immune function-related indicators; O4—adverse drug reactions; O5—quality of life KPS score; qd—once daily; tid—three times daily

### Methodological bias of the included studies

**Fig. 1:**
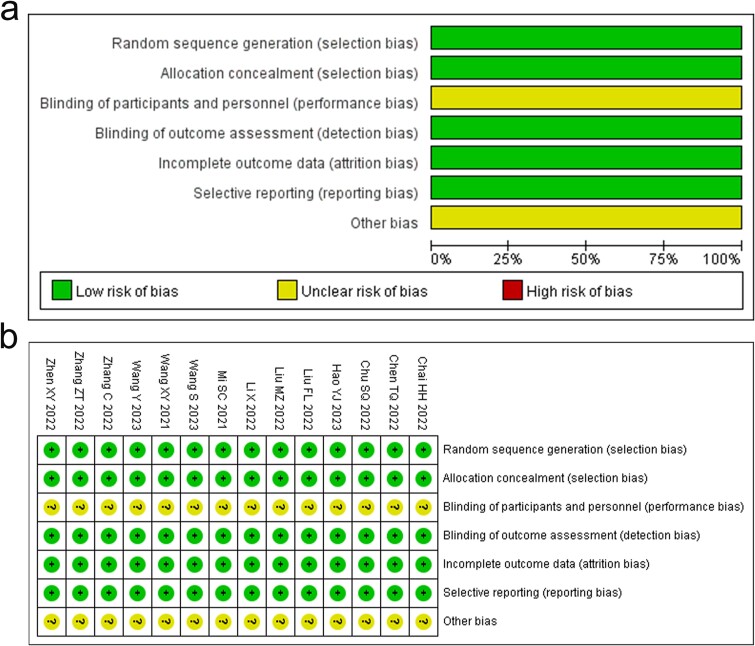
Risk of methodological bias of included studies. (a) Summary of bias risk: The review authors’ evaluation of each risk of bias factor, presented as a percentage for all papers included in the review. (b) Risk of bias graph: The review authors evaluated the risk of bias for each included study.

The technique of random allocation was explicitly outlined in all 14 experiments. All participants in the experiments were kept oblivious of their assigned group, however, it was not explicitly stated whether the doctors conducting the trials were also knowledgeable of this information. The trial data were comprehensive and there was no evidence of selective reporting in the trials. There were no additional documented prejudices. Figure 1 displays the attributes and excellence of all the trials that were considered.

### Tumor response

The National Cancer Institute (NCI) and the European Organisation for Research and Treatment of Cancer (EORTC) collaborated to create a standardized system called Response Evaluation Criteria in Solid Tumors (RECIST). The introduction of the RECIST criteria has served as a valuable evaluation standard for solid tumors. It has provided malignant tumor patients with precise reference indicators for diagnosis, treatment, and postoperative surveillance. Furthermore, the implementation of the RECIST criteria as a globally acknowledged assessment standard has facilitated enhanced communication and more efficient analysis among researchers worldwide. The criteria established by the World Health Organization (WHO): In 1979, the World Health Organization (WHO) organized seminars to establish a standardized system for reporting the outcomes of cancer treatment.^33^

Techniques were devised to gather information on patients, tumors, experimental and radiographic data, assessing the severity of acute/subacute toxicity, documenting the results of cancer treatment, and establishing uniform guidelines for reporting treatment outcomes. The RECIST criteria were initially introduced in 1999 at the ASCO conference in the United States and subsequently published in the JNCI (Journal of the National Cancer Institute) in the same year. The updated iteration of RECIST was initially released in 2009, incorporating appropriate adjustments and supplements to the preceding WHO criteria. The data sources are vast, and the criteria used are highly cited and standardized.^34^

**Fig. 2:**
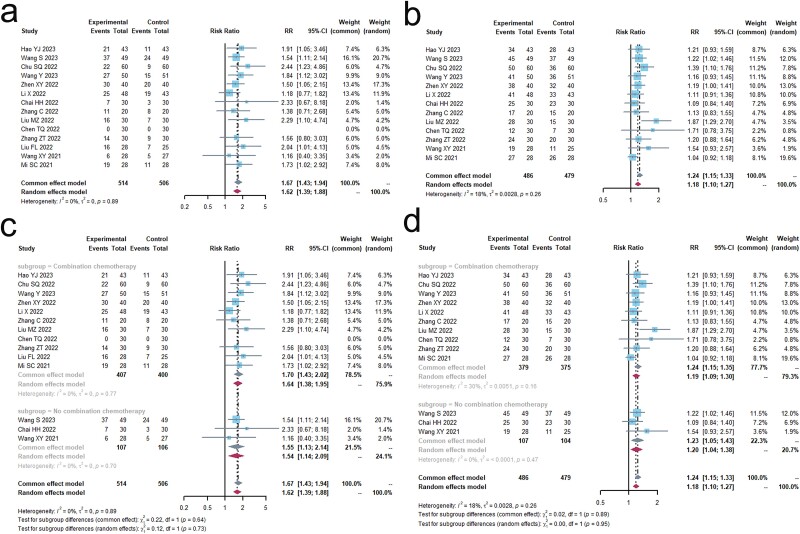
Short-term clinical efficacy. (a) Forest plot of ORR analysis results. (b) Forest plot of DCR analysis results. (c) Forest plot of ORR subgroup analysis results. (d) Forest plot of DCR subgroup analysis results.

As per the recommendations provided by the WHO^33^ or the RECIST^35^, a total of 14 trials including 1,020 patients were conducted to evaluate the objective response rate (ORR). Additionally, 13 trials^19^^,^^36–47^ involving 965 patients were conducted to analyze the DCR. These findings are presented in Fig. 2 and Table 2. The results of Cochrane’s Q test and I^2^ statistics indicated the absence of heterogeneity (ORR, I^2^ = 0.0%, *P* > 0.1; DCR, I^2^ = 17.7%, *P* > 0.1). When TCM was used together with PD-1/PD-L1 inhibitors, there was a substantial increase in the objective response rate (ORR) (relative risk [RR] 1.67 [1.43; 1.94], *P* < 0.0001) and disease control rate (DCR) (RR 1.24 [1.15; 1.33], *P* < 0.0001) compared to the control group.

**Table 2:** Results of meta-analysis of short-term clinical efficacy.

Outcomes	Trials	Combination therapy group	Control group	SM	RR,95% CI	I^2^ (%)	Q	p	PB
		(Events/Total)	(Events/Total)						
ORR	14	251/514	148/506	CEM	1.6661 [1.4296; 1.9416]	0.0	0.8866	< 0.0001	No
DCR	13	401/486	319/479	CEM	1.2398 [1.1535; 1.3326]	17.7	0.2647	< 0.0001	No

Note: Forest of all results are in Fig. 4

Abbreviations: CI, confidence interval; CEM, common effects model; PB, Publication bias; REM, random- effects model; RR, relative ratio; SM, statistical method

We conducted a meta-analysis of the ORR, dividing it into two subgroups: the combination chemotherapy group (*n* = 11) and the non-combination chemotherapy group (*n* = 3). Both subgroups showed no significant differences (I^2^ = 0.0%) and both groups demonstrated a significant improvement in the ORR. The combination chemotherapy group had a risk ratio (RR) of 1.70 [1.43; 2.02], *P* < 0.0001, while the non-combination chemotherapy group had an RR of 1.55 [1.13. 2.14], *P* < 0.0001.

We conducted a meta-analysis on DCR, dividing it into two subgroups: the combination chemotherapy group (*n* = 11, I^2^ = 30%) and the non-combination chemotherapy group (*n* = 3, I^2^ = 0.0%). Both subgroups were not heterogeneous, and both showed a significant improvement in DCR. The combination chemotherapy group had a relative risk (RR) of 1.24 [1.15; 1.35], *P* < 0.0001, while the non-combination chemotherapy group had an RR of 1.23 [1.05; 1.43], *P* < 0.0001.

### Tumor markers and related factors

**Fig. 3:**
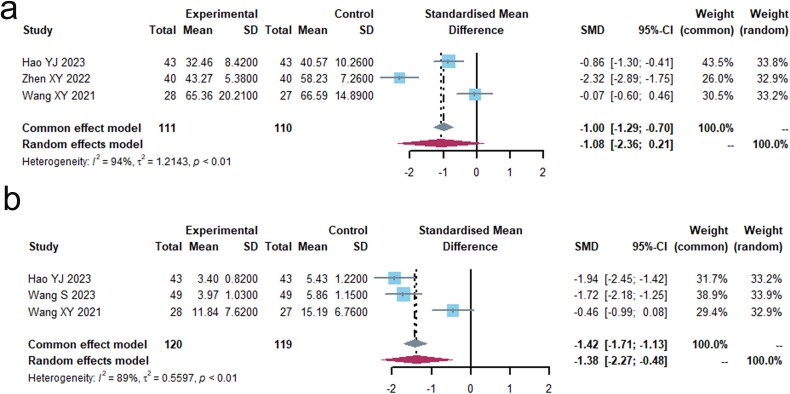
Tumour markers. (a) CA125. (b) CYFRA21-1.

Four investigations^19^^,^^36^^,^^37^^,^^40^ with a total of 319 individuals were examined for the presence of CA125 or CYFRA21-1. The results are shown in Fig. 3 and Supplementary Table 1. The I^2^ value indicated a high level of heterogeneity (I^2^ > 80%). The study results demonstrated a significant statistical difference in CYFRA21-1 levels between the two groups (standardized mean difference, −1.3754 [−2.2711; −0.4798], *P* < 0.05), whereas there was no significant statistical difference in CA125 levels (standardized mean difference, −1.0758 [−2.3580; 0.2063], *P* = 0.1001). In the four investigations, Glehniae Radix, Radix Astragali, Radix Ophiopogonis, and Rhizoma Atractylodis Macrocephalae were frequently used, which are strong in their effects of Nourishing Yin Sheng Jin and Invigorating spleen Replenishing Qi. The statistical results indicate that these drugs have a good effect in assisting the reduction of CYFRA21-1.

### The levels of peripheral blood lymphocytes

**Fig. 4:**
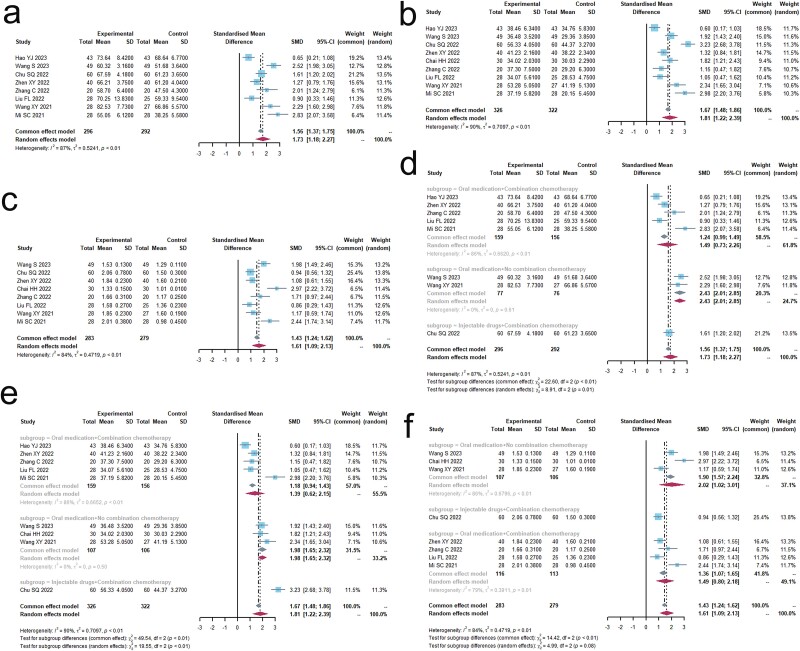
Immune function (a) Forest plot of CD3+ analysis results. (b) Forest plot of CD4+ analysis results. (c) Forest plot of CD4+/ CD8+ analysis results. (d) Forest plot of the results of CD3+ subgroup analysis. (e) Forest plot of the results of CD4+ subgroup analysis. (f) Forest plot of the results of CD4+/ CD8+ subgroup analysis.

A collective of 648 patients from 9 studies^19^^,^^36–38^^,^^40^^,^^42^^,^^43^^,^^47^^,^^48^ provided data on peripheral blood lymphocyte counts (Fig. 4, Supplementary Table 2). There were 3 diverse results with a high level of heterogeneity (I^2^ > 80%). The results indicated that the test group saw significant improvements in the proportion of CD3+ T cells (SMD, 1.7278 [1.1849; 2.2708], *P* < 0.0001), CD4+ T cells (SMD, 1.8057 [1.2210; 2.3903], *P* < 0.0001), and the ratio of CD4+/CD8+ T cells (SMD, 1.6079 [1.0885. 2.1272], *P* < 0.0001). In nine trials, Radix astragali appeared 6 times, ginseng, Glehniae Radix, and Radix ophiopogonis each appeared 3 times, serving as either principal drugs or adjuvant drugs. Furthermore, the formula also includes drugs such as Rhizoma Atractylodis Macrocephalae and Acanthopanacis Senticosi Radix.

We conducted an additional subgroup analysis, dividing the participants into three groups: one group receiving TCM in combination with PD-1/PD-L1 inhibitor and chemotherapy (*n* = 5, I^2^ = 86%). The combination of Glehniae Radix and Radix ophiopogonis appeared 2 times, and Radix astragali appeared 3 times. Ginseng and Rhizoma Atractylodis Macrocephalae appear twice. And another group receiving TCM paired with PD-1/PD-L1 inhibitor but not receiving chemotherapy (*n* = 2, I^2^ = 0.0%). The commonly contained medicines are Radix astragali, Rhizoma atractylodis Macrocephalae, and Ligustri Lucidi Fructus. The last group is made up of PD-1/PD-L1 inhibitors, chemotherapy, and traditional Chinese medicine injections that contain Ginseng, Radix Astragali, and Acanthopanacis Senticosi Radix. Significant improvement in CD3+ T cells was observed in all test groups.

CD4+/CD8+ T cells were divided into two groups: The first group consisted of TCM combined with PD-1/PD-L1 inhibitor plus chemotherapy, with a sample size of 4 and an I^2^ value of 79%. The second group consisted of TCM combined with PD-1/PD-L1 inhibitor, without chemotherapy, with a sample size of 3 and an I^2^ value of 86%. Significant improvement in CD4+/CD8+ T cells was observed in all test groups.

### Adverse drug reactions

**Fig. 5:**
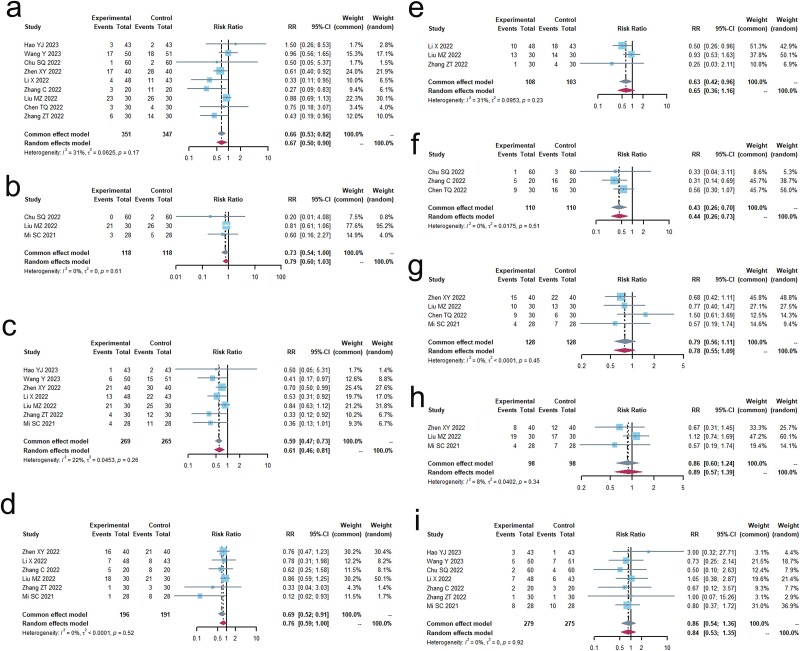
Adverse drug reactions. (a) Forest plot of the results of the nausea and vomiting analysis. (b) Forest plot of analytical results for diarrhoea. (c) Forest plot of the results of the leukopenia analysis. (d) Forest plot of the results of the platelet reduction analysis. (e) Forest plot of haemoglobin reduction analysis results. (f) Forest plot of fatigue analysis results. (g) Forest plot of hepatic impairment analysis results. (h) Forest plot of the results of the renal impairment analysis. (i) Forest plot of rash analysis results.

In 10 trials^36^^,^^38–41^^,^^43–47^, a total of 877 adverse events were reported. The results for nine of these adverse events were not heterogeneous, with an I^2^ value of less than 40% (Fig. 5, Table 3). Chemotherapy was used in conjunction with 10 trials.

**Table 3:** Results of a meta-analysis of adverse drug reactions.

Outcomes	Trials	Microecological preparation group	Control group	SM	RR,95% CI	I^2^ (%)	Q	p	PB
		(Events/Total)	(Events/Total)						
Nausea and vomiting	9	77/351	116/347	CEM	0.6609 [0.5345; 0.8173]	31.4	0.1667	<0.0001	No
Skin rash	7	28/279	32/275	CEM	0.8576 [0.5394; 1.3634]	0.0	0.9211	0.5159	No
Leukopenia	7	70/196	117/191	CEM	0.6857 [0.5180; 0.9077]	0.0	0.5212	0.0084	No
Thrombocytopenia	6	48/196	69/191	CEM	0.6857 [0.5180; 0.9077]	0.0	0.5212	0.0084	No
Reduced haemoglobin	3	24/108	36/103	CEM	0.6340 [0.4171; 0.9637]	31.2	0.2337	0.0329	No
Hepatic impairment	4	38/128	48/128	CEM	0.7917 [0.5629; 1.1134]	0.0	0.4542	0.1794	No
Renal impairment	3	31/98	36/98	CEM	0.8611 [0.5979; 1.2402]	8.5	0.3354	0.4218	No
Fatigue	3	15/110	35/110	CEM	0.4286 [0.2636; 0.6969]	0.0	0.5107	0.0006	No
Diarrhoea	3	24/118	33/118	CEM	0.7313 [0.5366; 0.9968]	0.0	0.6107	0.0477	No

The results showed a significant statistical difference in the occurrence of nausea and vomiting (RR, 0.6609 [0.5345; 0.8173], *P* < 0.0001), leucopenia (RR, 0.6857 [0.5180; 0.9077], *P* < 0.05), decrease in hemoglobin levels (RR, 0.6341 [0.4171; 0.9637], *P* < 0.05), thrombocytopenia (RR, 0.6857 [0.5180; 0.9077], *P* < 0.05), fatigue (RR, 0.4286 [0.2636; 0.6969], *P* < 0.05), and diarrhea (RR, 0.7313 [0.5366; 0.9968], *P* < 0.05). In 10 trials, Radix Astragali appeared 4 times, the combination of Radix Astragali and Ginseng appeared 2 times, and the combination of Radix Astragali and Rhizoma Atractylodis Macrocephalae appeared 2 times. Glehniae Radix appeared 3 times, and the combination of Glehniae Radix and Radix Ophiopogonis appeared 2 times.

The findings did not show any statistically significant differences in the occurrence of rash (RR, 0.8576 [0.5394; 1.3634], *P* = 0.5159), liver dysfunction (RR, 0.7917 [0.5629; 1.1134], *P* = 0.1794), and renal dysfunction (RR, 0.8611 [0.5979; 1.2402], *P* = 0.4218). Among these, the combination of Radix Astragali and Rhizoma Atractylodis Macrocephalae appeared twice, the combination of Glehniae Radix and Radix Ophiopogonis appeared twice, and the combination of Ginseng and Radix Astragali appeared twice.

### Quality of life

**Fig. 6:**
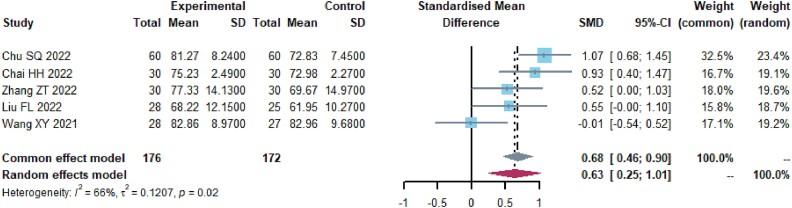
Forest plot of the results of the KPS score analysis.

A combined total of 348 patients from five studies^19^^,^^38^^,^^42^^,^^46^^,^^48^ provided KPS scores, as shown in Fig. 6 and Supplementary Table 3. The results exhibited heterogeneity, as shown by an I^2^ value of 66.5% which is greater than the threshold of 50%. The findings indicated that the research group exhibited a significant improvement in patients’ KPS scores (standardized mean difference [SMD] of 0.6296 [0.2514; 1.0077], *P* < 0.01). Among them, Radix Astragali appeared 4 times, the combination of Radix codonopsis, Radix Astragali, and Rhizoma Atractylodis Macrocephalae appeared 2 times, and Ginseng appeared 2 times.

### Publication bias analysis

The primary purpose of the funnel plot is to assess potential bias in the results of a meta-analysis. For practical purposes, it is typically advised that a meta-analysis aiming to create a funnel plot should comprise a minimum of 10 or more study volumes. Since both the ORR and DCR investigations consisted of more than 10 individual studies, we created a graphical representation known as a funnel plot.

The dots in the diagram show the studies that have been included. Statistical sampling theory suggests that increasing the sample size leads to more dependable results, reduced variability, smaller standard error, and a higher concentration of data points in the narrower top area of the funnel plot. Conversely, a smaller sample size results in increased variability, larger variance, greater standard error, and scattered data points in the wider bottom area of the funnel, resulting in an inverted funnel shape. The arrangement of the dots in the diagram indicates that the overwhelming majority of the research we considered possess substantial sample sizes. Nevertheless, we did not eliminate a few trials with smaller sample sizes that include comprehensive descriptions of the intervention process and complete outcome data.

**Fig 7:**
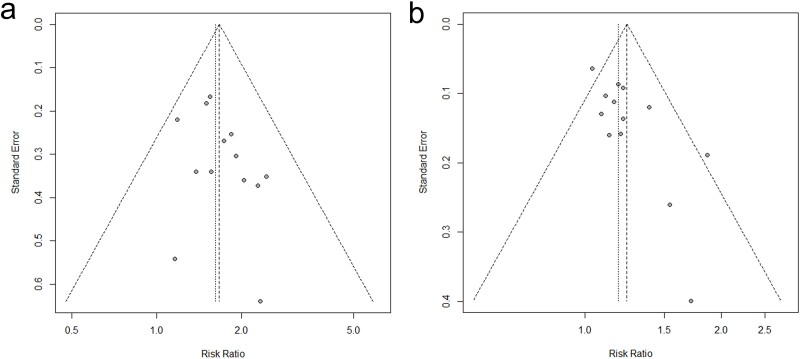
Publication bias analysis. (a) ORR. (b) DCR.

The distribution of studies in the funnel plot exhibits approximate symmetry, indicating the absence of publishing or other biases in the research (Fig. 7).

### Sensitivity analysis

**Fig. 8:**
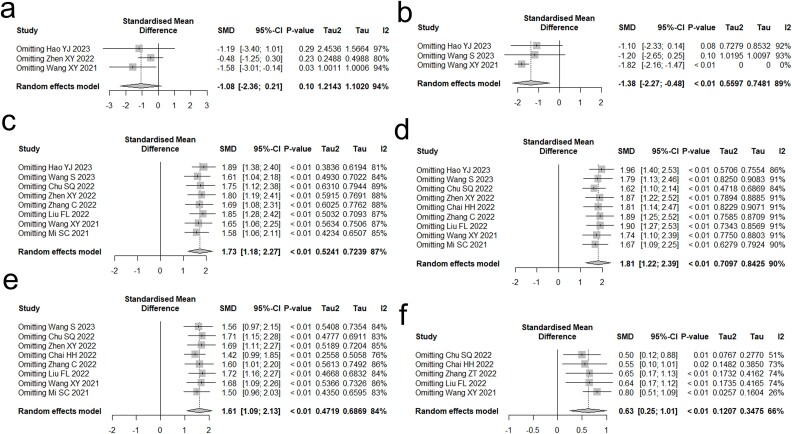
Sensitivity analysis. (a) CA125. (b) CYFRA21-1. (c) CD3+. (d) CD4+. (e) CD4+/CD8+. (f) KPS.

Sensitivity analyses involve conducting statistical analyses multiple times, using different assumptions and methodologies, in order to assess the impact on the results and determine the amount of any changes. The objective of sensitivity analysis is not to identify the most advantageous outcomes, but rather to assess the robustness of the initial findings. Consequently, we conducted a meta-analysis by removing one publication at a time and analyzed the impact of these exclusions on the overall results. This allowed us to determine if the initial meta-analysis results were significantly affected by the inclusion or exclusion of specific studies. If the outcomes of the sensitivity analyses were congruent with the outcomes of the main analyses, it signified that the present conclusions were resilient. (Fig. 8).

### Subgroup analysis of general information

The age, sex, and histological type of tumor of the patients included in the study in the experimental and control groups did not show any statistically significant differences (*P* > 0.05) and were similar. (Supplementary Fig. 2.).

## Discussion

This evaluation includes 1,020 patients from 14 randomized controlled clinical studies to evaluate the clinical effectiveness and safety of nourishing yin and replenishing qi traditional Chinese medicine (TCM) with PD-1/PD-L1 inhibitors in the treatment of non-small cell lung cancer. The study findings demonstrated that the treatment group exhibited greater rates of ORR and DCR compared to the control group. Subgroup analysis further confirmed these outcomes. This outcome holds significant importance for individuals with cancer who are currently receiving medical therapy. As part of the study, we conducted a comparison of the quality of life and peripheral blood T-lymphocyte counts in each group. The expression of T lymphocytes was linked to a negative prognosis and the spread of tumors. The findings indicated that the use of TCM treatments that nourishing yin and replenishing qi, administered orally or through injections, in combination with PD-1/PD-L1 inhibitors, were successful in enhancing the percentage of CD3+ T cells and CD4+ T cells. Additionally, this treatment improved the CD4+/CD8+ T cell ratio and the overall quality of life.

Furthermore, the findings indicated that the integration of nourishing yin and replenishing qi TCM with PD-1/PD-L1 inhibitors resulted in a decrease in the expression of the tumor marker CYFRA21-1. Cytokeratin 19 fragment (CYFRA21-1) is a protein that plays a crucial role in maintaining the structure of monolayer epithelial cells. It can be found in serum endothelial cells and indicates the death and breakdown of tumor cells in tumor cytopathies^49^. CYFRA21-1 levels can be used to assess the prognosis of patients with NSCLC, and consistently high levels of CYFRA21-1 suggest a negative prognosis for NSCLC patients^50^. Administering a combination of nourishing yin and replenishing qi TCM alongside PD-1/PD-L1 inhibitors can potentially decrease the occurrence of adverse effects such as nausea, vomiting, diarrhea, leukopenia, thrombocytopenia, hemoglobin reduction, and malaise in patients. Moreover, this combination treatment does not impose additional strain on liver and kidney function.

PD-1 and PD-L1 play crucial roles as inhibitory factors in the regulation of the immune system^51^^,^^52^. Tumor cells exhibit high levels of PD-L1 expression on their cell surface. Tumor cells have the ability to attach to PD-1 receptors on the surface of T cells by using their PD-L1 receptors. This attachment inhibits the activity of T cells through internal signaling, which ultimately weakens the immune response against tumor cells and makes them resistant to traditional radiotherapy^53^^,^^54^. The PD-1/PD-L1 axis is crucial in controlling T cell immunity and has been linked to both autoimmune and infection^55^. PD-L1 expression in NSCLC is linked to heightened tumor cell proliferation and invasiveness, as well as reduced patient survival^56^. The interactions between PD-1 and PD-L1 are referred to be “immune checkpoints” due to their role in regulating the immune response to tumor antigens. China’s National Medical Products Administration has granted approval for several immunotherapy drugs, including Pembrolizumab, Camrelizumab, Atezolizumab, Sintilimab, Tislelizumab, and Nivolumab, to be used in the treatment of metastatic non-small cell lung cancer. These approvals have been granted since the beginning of 2019. Camrelizumab, Tislelizumab, and Sintilimab are Chinese-developed PD-1 inhibitors.

We incorporated a total of 14 trials, which encompassed five PD-1/PD-L1 inhibitors: Atezolizumab, Pembrolizumab, Camrelizumab, Nivolumab, and Sintilimab. The procedures adhered to the Chinese clinical practice guidelines for treating NSCLC.

It is important not to overlook the presence of irAEs, as they restrict the effectiveness of medical treatment and, in more serious instances, put patients’ lives at risk^57^. Consequently, current research is focused on ways to enhance the effectiveness of ICIs and minimize their adverse effects for the benefit of patients. Contemporary medicine holds the belief that compromised immune function plays a significant role in the onset and progression of malignant tumors. Traditional Chinese medicine posits that the development of accumulation is caused by a lack of positive qi and the proliferation of evil qi. This aligns with the contemporary concept of tumor immunity in the field of modern oncology^58^. Consequently, the integration of PD-1/PD-L1 inhibitor therapy with other pharmacological regimens to enhance the adverse effects of PD-1/PD-L1 inhibitors has emerged as a prominent area of focus in tumor therapy.

Chinese anti-tumor medicine possesses the qualities of being comprehensive, systematic, and multi-faceted. It has the ability to enhance the internal environment of the human body and stimulate the autoimmune system to combat tumors by strengthening the body’s immunological function^25^^,^^26^. This notion bears resemblance to the concept of utilizing immunotherapeutics for the treatment of malignancies. By utilizing a combination of Traditional Chinese Medicine (TCM) therapeutic drugs that reduce toxicity and enhance sensitization, it is feasible to enhance patients’ responsiveness to immunotherapy, enhance the clinical effectiveness of immunotherapy, minimize adverse effects, and extend patients’ survival. In Traditional Chinese Medicine (TCM) theory, the primary manifestation of immunity is referred to as “righteousness,” which aligns with the concept of immunotherapy in contemporary medicine^23^^,^^24^. The implementation of a PD-1 monoclonal antibody has substantially increased the longevity of individuals diagnosed with lung cancer^59^^,^^60^. The PD-1 monoclonal antibody improves the efficacy of T lymphocytes in eliminating tumor cells by stimulating the response of T lymphocytes towards tumor cells^61^^,^^62^. This aligns with the principles of Chinese medicine, which emphasize identifying the underlying cause of disease and the notion that a strong internal state can prevent external disturbances.

Once cancer develops, it persists for an extended period, depleting the body’s healthy Qi, fluids, and humor, hence worsening the Qi-Yin insufficiency pattern. An examination of the frequency and correlation of Chinese medicine symptoms in a substantial sample of 7,435 cases of non-small cell lung cancer (NSCLC) revealed that qi deficit and yin deficiency are the fundamental symptoms of NSCLC^63^. Professor Zhou Zhongying, an expert in Chinese medicine, states that lung cancer is mostly caused by a lack of good qi and a deficiency of both qi and yin^64^. Wang Ji formulated the concept of “Ying Wei Yi Qi,” which states that Radix Ginseng and Radix Astragali have the ability to nourish both qi and yin. Additionally, he promoted the idea of “ShenQi ShuangBu,” which laid the groundwork for strengthening and nurturing yin. The majority of contemporary Chinese medicine practitioners assert that the primary cause of middle- and late-stage non-small cell lung cancer (NSCLC) is a shortage of qi and yin. They advocate for the approach of enhancing qi and nourishing yin in the treatment of NSCLC.^65^

Regarding the molecular mechanisms of traditional Chinese medicine for replenishing Qi and nourishing Yin in the treatment of NSCLC. On one hand, they can directly kill tumor cells. Traditional Chinese medicine contains various active components, such as polysaccharides, alkaloids, and flavonoids. These can block the cell cycle, inhibit tumor cell proliferation, suppress tumor cell DNA synthesis, promote tumor cell apoptosis, and specifically target the growth signaling pathways of tumor cells.^66–69^ Ginsenoside Rg3 induces apoptosis in lung cancer cells through various signaling pathways such as PI3K/Akt, NF- κB, EGFR, and FUT4/LeY.^70–73^ At the same time, Rg3 can regulate the expression of PD-1 and PD-L1 in NSCLC cells, blocking the interaction between PD-1 and PD-L1.^74^ Total saponins from lilies are one of the compounds extracted from lilies. They can reduce the Bcl-2/Bax protein ratio in lung cancer cells, activate the apoptosis factor Pro-caspase3, and induce apoptosis in cancer cells.^75^ On the other hand, traditional Chinese medicine for replenishing Qi and nourishing Yin can act on the immune cells of the human body, participate in anti-tumor immune responses, reverse immune evasion in lung cancer, inhibit tumor cell metastasis, and achieve the goal of suppressing NSCLC.^76^ The inhibitory effect of the Spleen-Invigorating and Phlegm-Resolving Decoction on tumors in patients with Spleen Qi deficiency and phlegm-dampness type non-small cell lung cancer may be related to the reduced mRNA expression of NF-kB signaling molecules and their downstream products by this formula.^77^ Astragaloside can prevent immune evasion events and inhibit the spread of NSCLC cancer cells, possibly related to the suppression of the ILT4-PI3K/Akt-B7-H3 pathway.^78^ Astragalus polysaccharides can reduce CA125 levels and VEGF expression in NSCLC patients, and when used concurrently with radiotherapy, they can decrease the incidence of bone marrow suppression.^79^ Ginsenoside Rg3 can protect DNA integrity by activating the VRK1/P53BP1 pathway, thereby inhibiting tumorigenesis and activity in NSCLC cells.^80^ Glehniae Radix, Radix ophiopogonis, and greenish lily bulb can inhibit tumor metastasis by regulating adhesion, matrix degradation, and VEGFR expression during the tumor metastasis process.^81^ Ginsenosides, glycyrrhizin, and Polygonatum polysaccharides can enhance the activity of T cells, NK cells, and macrophages by intervening in signaling pathways such as P53, thereby improving the body’s ability to monitor and eliminate tumor cells.^82^ Finally, they can inhibit tumor angiogenesis. For example, ginsenosides can significantly inhibit tumor angiogenesis by suppressing the activity of vascular endothelial growth factor (VEGF), thereby cutting off the tumor’s nutrient supply and inhibiting tumor growth.^83^

Regarding the current issues with TCM for combination therapy, first of all, the current research mostly consists of clinical study results with small sample sizes, minimal regional differences, and short observation periods, making it difficult to reflect the “tail effect” of immune checkpoint inhibitors, which is insufficient to represent the long-term clinical benefits for patients. In the future, we need more multicenter, randomized, prospective, long-term observational, large-sample clinical trials to obtain objective data to confirm the long-term clinical effects of traditional Chinese medicine combined with chemotherapy in the treatment of advanced NSCLC. Secondly, the non-standardized and personalized characteristics of TCM treatment somewhat limit its promotion and application. TCM emphasizes the dialectical thinking of the “holistic concept,” and the lack of unified dialectical differentiation and evaluation standards in clinical practice is not conducive to the development of related research. In the future, authoritative experts should be organized to formulate efficacy evaluation standards for the treatment of symptoms related to advanced non-small cell lung cancer, fully utilizing the combined advantages of traditional Chinese medicine and Western medicine. This will standardize the formulation of plans for integrated Chinese and Western medicine treatment at various stages, making it easier for grassroots Chinese medicine service institutions to better evaluate and choose different therapies. Finally, the research and development of drugs are lagging behind, and there is still a lack of in-depth studies on the intrinsic molecular mechanisms and targets of traditional Chinese medicine in treating NSCLC, especially in relation to immune-related research. Therefore, in the future, we should intensify research and development efforts to screen for effective components and drugs with anti-cancer properties. At the same time, research directions should further clarify the mechanisms by which they treat NSCLC, delving deeper and broader to achieve a more perfect integration of traditional Chinese and Western medicine.

## Conclusions

The results of our study demonstrate that the integration of nourishing yin and replenishing qi traditional Chinese medicine with PD-1/PD-L1 inhibitors significantly enhances the clinical effectiveness and safety of NSCLC treatment. Additionally, the combination of Chinese and Western medicine improves the efficacy of neoadjuvant chemotherapy in NSCLC patients.

## Supplementary Material

Supplementary_materials_tfaf013

## Data Availability

Data sharing is not applicable to this article as no new data were created or analyzed in this study.
