# Supplementary material for: Clinical efficacy and safety evaluation of traditional Chinese medicine for nourishing yin and Replenishing qi in combination with PD-1/PD-L1 inhibitors in the treatment of NSCLC patients: a meta-analysis
Source: Toxicol Res (Camb). 2025 Jan 26;14(1):tfaf013. doi: 10.1093/toxres/tfaf013 (PMC11766746; doi:10.1093/toxres/tfaf013)
Supplement: Supplementary_materials_tfaf013 [file supplementary_materials_tfaf013.docx]

**Supplementary materials**


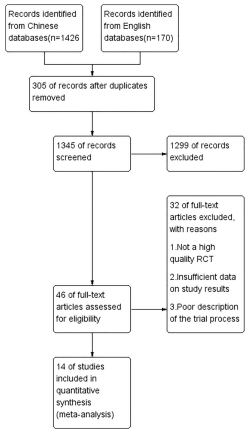


**Supplementary Figure 1.** The flow charts of included studies

A(Age)
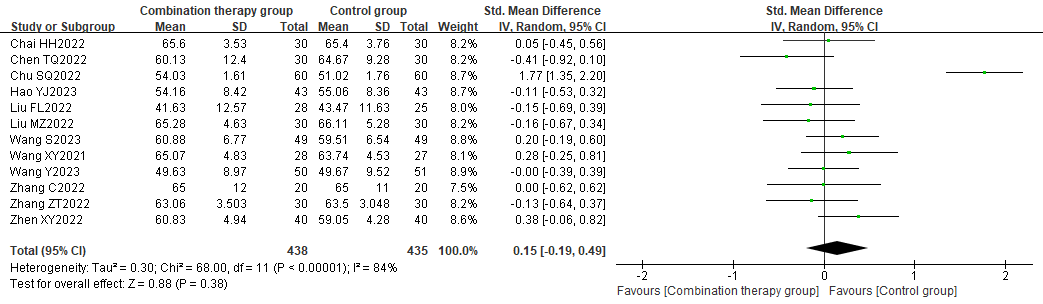


B (Sex)


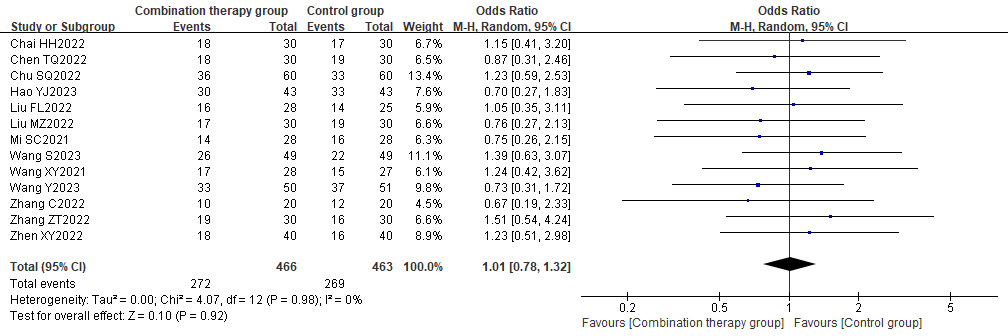


C (Histological Type)


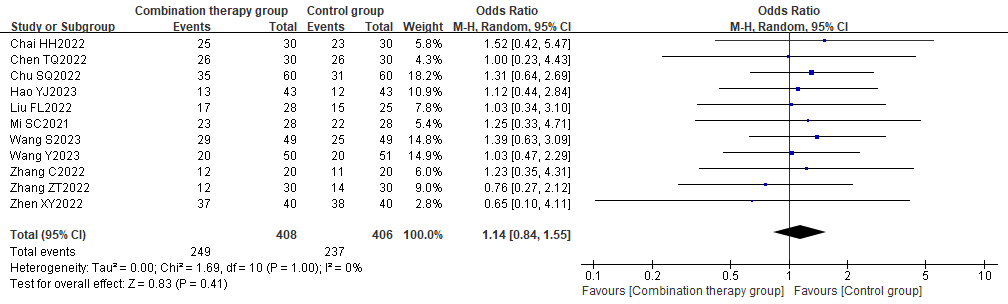
**Supplementary Figure 2.** Subgroup analysis of age, sex, and histological typing.

**Supplementary Table 1** Results of meta-analysis of tumour markers

| **Outcomes** | **Trials** | **SM** | **SMD,95% CI** | **I^2^ (%)** | **Q** | **p** | **PB** |
| --- | --- | --- | --- | --- | --- | --- | --- |
| **CA125** | 3 | REM | -1.0758  [-2.3580; 0.2063] | 93.9 | < 0.0001 | 0.1001 | Yes |
| **CYFRA21-1** | 3 | REM | -1.3754  [-2.2711; -0.4798] | 88.8 | 0.0001 | 0.0026 | Yes |

**Supplementary Table 2** Results of meta-analysis of immune function-related indicators

| **Outcomes** | **Trials** | **SM** | **SMD,95% CI** | **I^2^ (%)** | **Q** | **p** | **PB** |
| --- | --- | --- | --- | --- | --- | --- | --- |
| **CD3^+^** | 8 | REM | 1.7278  [1.1849; 2.2708] | 86.6 | < 0.0001 | < 0.0001 | Yes |
| **CD4^+^** | 9 | REM | 1.8057  [1.2210; 2.3903] | 89.9 | < 0.0001 | < 0.0001 | Yes |
| **CD4^+^/CD8^+^** | 8 | REM | 1.6079  [1.0885; 2.1272] | 83.7 | < 0.0001 | < 0.0001 | Yes |

**Supplementary Table 3** Results of meta-analysis of KPS scores

| **Outcomes** | **Trials** | **SM** | **SMD,95% CI** | **I^2^ (%)** | **Q** | **p** | **PB** |
| --- | --- | --- | --- | --- | --- | --- | --- |
| **KPS** | 5 | REM | 0.6296  [0.2514; 1.0077] | 66.5 | 0.0178 | 0.0011 | Yes |
